# Relationship between serum nitric oxide of patients with thyroid disorders and metabolic syndrome indices and nitrate concentration of water

**DOI:** 10.1038/s41598-023-27560-0

**Published:** 2023-01-13

**Authors:** Shaghayegh Jafari, Mansooreh Dehghani, Haleh Ghaem, Mahmood Soveid, Hasan Hashemi

**Affiliations:** 1 Department of Environmental Health Engineering, School of Health, Student Research Committee, Shiraz University of Medical Sciences, Shiraz, Iran; 2 Research Center for Health Sciences, Department of Environmental Health, School of Health, Shiraz University of Medical Sciences, P.O.Box: 111, Shiraz, 71645 Iran; 3 Department of Epidemiology, Non-Communicable Diseases Research Center, School of Health, Shiraz University of Medical Sciences, Shiraz, Iran; 4 Endocrinology Research Center, Nemazee Hospital, Shiraz University of Medical Sciences, Shiraz, Iran; 5 Research Center for Health Sciences, Institute of Health,Department of Environmental Health Engineering, School of Health, Shiraz University of Medical Sciences, Shiraz, Iran

**Keywords:** Diseases, Health care

## Abstract

This case–control study aimed to assess the effect of drinking water nitrate on serum nitric oxide concentration and the risk of metabolic syndrome (MetS) in the population in the Middle East. The study included 50 control and 50 thyroid disorder cases who were referred to two medical centers in 2021. In this study, serum nitric oxide concentration, drinking water nitrate, and metabolic syndrome components were measured in the two groups. The results showed there was a statistically significant difference between serum NO in the case and control groups (p-value < 0.001). There was a positive correlation between the concentration of nitrate in drinking water and serum nitric oxide in the case and control groups; however, this relationship was not significant statistically. A statistically significant difference was found between serum nitric oxide and systolic blood pressure in the cases (p-value < 0.05), but there was no significant difference between MetS and nitric oxide. Therefore, we concluded that the relationship between nitric oxide and nitrate in consuming water should be determined in thyroid patients. In addition to their water consumption, it is better to study the nitrate of foods, especially vegetables.

## Introduction

Nitrate and nitrite levels are currently increasing due to the excessive use of fertilizers and industrial development^1^. Nitrate is a common contaminant in drinking water, especially in agricultural areas where the use of nitrogen fertilizers has increased its concentration in drinking water sources since the 1950s^2–4^. In addition to agriculture, leachate from the waste accumulation and pollution due to human and animal wastes cause a considerable increase in nitrate concentration in the surface and groundwater^5^.

Nitrate is unstable in the human body and metabolized by human enzymes. Nitrate reduction activities by bacteria may convert nitrate to nitrite and other nitrogen compounds, which affects the physiological state and human health. After consumption, nitrate absorbed by the upper gastrointestinal tract easily. More than 25% of it is excreted into the saliva, and 20% is transformed to nitrite by oral bacteria^6^. Under acidic conditions in the stomach, nitrite is altered to nitrogen trioxide (N_2_O_3_), nitric oxide (NO), and nitrogen dioxide (NO_2_) as the nucleus of nitric acid (HNO_2_)^7^. Nitric oxide is a lipophilic molecule that is involved in various physiological and pathophysiological processes^8,9^ and is also the first gas molecule to be described as a mediator in signaling pathways^10^. Recent evidence suggests that NO is involved in the adjustment of thyroid function^11^ and perhaps in the thyroid arteries and blood flow adjustment^12^. Nitric oxide (NO), hydroxyl radical (OH), superoxide anion (O_2_^−^), and hydrogen peroxide (H2O2) are free radicals that are considered oxidative stress^13^. Oxidative stress is a general term used to describe the state of damage caused by ROS (reactive oxygen species)^14^. ROS has a high reaction potential and will be toxic and lead to oxidative damage to cellular macromolecules such as proteins, lipids, and DNA^15^. Recently, the possible association between thyroid dysfunction and reactive oxygen species has been increasingly considered^16^. High doses of nitrate can also inhibit iodine uptake and cause hypertrophic changes in the thyroid gland^13^. Decreased iodine intake can lead to lower production of thyroid hormones T3 and T4 and thus increase the production of thyroid-stimulating hormone (TSH)^14^. The association of thyroid disease with the risk of metabolic syndrome (hypertension, glucose, HDL, triglyceride, and waist circumference) has been reported in several studies^15–24^. Diet quality is also a factor influencing the development of MetS due to diet-related inflammatory processes associated with metabolically unhealthy obesity (MUO) and metabolic syndrome (MetS)^25^.

Various studies have shown that the prevalence of MetS is about 15/5% in China and 35% in the United States^26^. The corresponding value in Iran has been reported in about 25% of the adult population^27,28^.

Mets is a chronic disease that, in addition to thyroid disease, increases the risk of many diseases, including cardiovascular disease, stroke, and type 2 diabetes. Mets can be diagnosed based on at least three of the following five conditions: High blood sugar (glucose levels ˃100 mg/dL), hypertension (systolic blood pressure (SBP) ≥ 130 mm Hg), and diastolic blood pressure (DBP) ≥ 85 mmHg abdominal obesity (waist circumference (WC) ˃ 102 and 88 cm in men and women, respectively), high serum triglycerides (TG levels ˃ 150 mg/dL), and lipoprotein cholesterol with High density (HDL-C < 50 and 40 mg/dL for men and women, respectively)^29^.

This case–control study aimed to assess the effect of drinking water nitrate on the serum nitric oxide concentration and the risk of metabolic syndrome (MetS) in the general population.

In recent years, diseases related to the thyroid gland in Shiraz have increased, and Fars province is a leader in agriculture. According to the research, the relationship between drinking water nitrate and serum nitric oxide has not been studied in Iran; therefore, we aimed to carry out this study due to the importance of drinking water for human health and its possible effect on thyroid.

## Materials and methods

### Study population, design, and sampling

This case–control study was performed on the population in Shiraz in 2021. Shiraz is the capital of Fars province (29° 36′ 37′′ North and 52° 31′ 52′′ East; 1486 m above sea level) and the fifth metropolis. Shiraz has a population of 1,869,000 people according to the 2016 census and is about 17,889 ha.

In recent years, the concentration of nitrate in Shiraz water has increased over the years due to various factors such as human processes, lack of proper hygiene, and placement of wells in groundwater; in some wells, it causes more nitrate than the standard (50 mg/L)^30,31^. Therefore, due to the relationship between nitrate and this disease^38^ and the increasing prevalence of thyroid disorders in this city^32^, this study was performed. Thyroid disorders include generalized goiter, thyroid nodules, hyperthyroidism or clinical hypothyroidism, and abnormal thyroid function in thyroid individuals^33^.

In this study, we looked at people with hypothyroidism, hyperthyroidism, and benign thyroid cancer as patients with thyroid disorders. In recent years, the amount of iodine has been sufficient in the study area, and the increase in the prevalence of hypothyroidism is due to increasing age^34^. T4 and TSH tests were also taken to determine if the control group had hormonal problems. 150 national codes of Shiraz residents were randomly selected, and their contact numbers were provided to us. The selected individuals were then contacted and 100 of them (including 50 controls and 50 cases) were referred to the medical diagnostic laboratory for the relevant tests on a specific day.

Inclusion criteria for the cases were: (a) fasting time (12–14 h), (b) people with thyroid disorders for at least one year, (c) consumption of piped municipal water as drinking water, and (d) residents of Shiraz.

Inclusion criteria for the controls were: (a) fasting time (12–14 h), (b) consumption of piped municipal water as drinking water, (c) residents of Shiraz, and (d) lack of a disease with Thyroid disorders. Informed consent was signed by the participants before taking the samples. Additionally, this study was approved by the Ethics Committee of Shiraz University of Medical Sciences (IR.SUMS.REC.1400.137), and The study followed the guidelines of the Declaration of Helsinki along with relevant guidelines^35^.

### Blood sampling to measure serum nitric oxide

In this study, to measure serum nitric oxide, after sampling the blood of case and control groups with a thyroid disorder, we left the gelled tubes containing their blood for at least 15 min and at most 30 min at ambient temperature (15–25 °C).

Then, they were centrifuged at 2500–3000 rpm for 10–15 min. The serum was separated from the tubes and stored at −20 °C for testing with each identification code. Nitric oxide was measured using a nitric oxide measuring kit (Kiazist Company, Iran).

In this experiment, Griess^39^ reagent was used as a reactant with nitric oxide. Nitric oxide was then bonded with a reagent and read at 540–560 nm^36^.

### Water nitrate assessment

After contacting the people to go to the laboratory, they were asked to bring a sample of drinking water in a bottle. Labeled samples in plastic bottles were transferred to the laboratory of the Department of Environmental Health of Shiraz University of Medical Sciences for chemical analysis.

In the first 24 h, the nitrate concentration of the samples was measured by the standard method using a spectrophotometer (UV (DR 6000) model) (hack company in Germany) at a wavelength of 220 nm for the samples^37^. Mean and standard deviation (SD) were calculated by SPSS Statistics 26.

### Assessment of metabolic syndromes

Concurrent with the measurement of blood samples, the participants' metabolic syndrome (blood pressure, waist circumference, glucose, HDL, and triglyceride) was measured. Also, age, sex, and amount of vegetables and fruits consumed in the samples (case and control groups) were asked daily and weekly. Anthropometric measurements including weight, height, and WC were also performed on the day of sampling.

Weight and height were measured according to the National Health and Nutrition Survey (NHANES) with minimal clothing and no shoes^38^. Blood pressure in the right hand was measured after ten minutes of rest (OMRON, M2 Japan). It was measured twice with an interval of ten minutes; then, the mean final blood pressure was considered. Blood biochemical parameters, including TG, HDL-C, and fasting blood sugar (FBS), were measured by an automated biochemical analyzer (Poch-100i, Sysmex, Kobe, Japan) in the clinical laboratory. Finally, Mets of risk were assessed based on NCEP-ATP III criteria^29^. The use of this criteria is preferred in Asia because they can be easily applied to primary care settings in many of those regions^39^.

### Statistical analysis

SPSS version 26 was used for all analyses. Mean and standard deviation were used to describe the quantitative variable. Pearson correlation coefficient was used to measure the correlation between variables if the data distribution was normal and the Spearman correlation coefficient was used if it was not normal. Linear regression was used to compare the correlation coefficients between the two groups.

**Figure 1:**
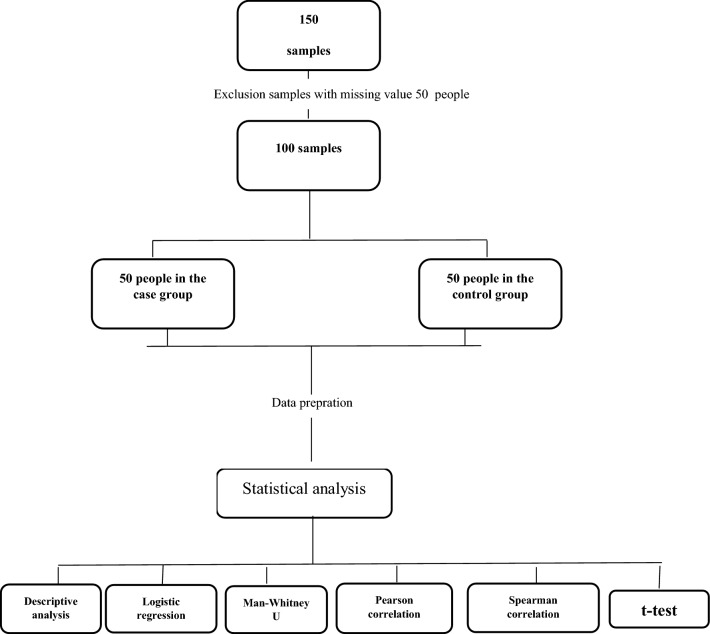
Study execution process.

Logistic regression was used to compare the metabolic syndrome between the two groups. To determine the relationship between normal variables of the body mass index, we used triglyceride from the independent t-test, and for the rest of the table data due to abnormality, Mann–Whitney test was used. Also, a duplicate test was used to the accuracy of the device and random error to measure serum nitric oxide and nitrate. To do this, we tested the samples twice in a row without a time interval. The process of conducting the study is shown in Fig. 1.

## Discussion and results

### Characteristics of the participants

According to Table 1, the mean age of the control group was 37.88 years; of them, 12 (24%) were male and 38 (76%) were female; also, for the cases, the mean age was 46.06 and among them 6 (12%) were male and 44 (88%) were female According to NCEP-ATP III criteria, the prevalence of MetS was 34% in the control group and 46% in the patient group with a thyroid disorder, which did not show a significant difference between the two groups (p value > 0.05) (Table 2).

**Table 1:** Characteristics of the participants.

Specifications	Demographic data for case group	Demographic data for control group
Age (year, mean ± SD)	11.73 ± 46.06	16.66 ± 37.88
Gender (female/male, number,%)	44.6 (88.12)	38.12 (76.24)
Consumption of vegetables (daily, weekly, number, %)	17.33 (34.66)	24.26 (48.52)
Frequency of fruit consumption (daily, weekly, number, %)	31.19 (62.38)	24.26 (48.52)
Metabolic syndrome (yes, no, number%)	23.27 (46.54)	17.33 (34.66)

**Table 2:** Serum NO and drinking water NO_3_ distribution levels in the case and control groups.

Parameters	Max	Min	SD ± Mean	Percentiles					P-value*
				10	25	50	75	90	
Serum NO concentration in case group	7.57	6.99	0.14 ± 7.26	7.05	7.14	7.26	7.34	7.47	≥ 0.001
Serum NO concentration in control group	7.44	6.97	0.12 ± 7.14	7.01	7.05	7.11	7.22	7.35	
NO_3_ concentration of drinking water in case group	47.5	3	13.92 ± 21.43	4.52	9.00	17.75	34.37	41.10	0.4
NO_3_ concentration of drinking water in the control group	45.5	3	14 ± 20.46	4.05	6.87	15.55	32.75	40.50	

*Man-Whitney U.

### Distribution of serum NO and nitrate levels in drinking water in the case and control groups

Table 3 shows the distribution of serum NO levels separately in the case and control groups.

**Table 3:** Metabolic syndrome components in the case and control groups.

Clinical examinations	Case			Control			P- value
	Max	Min	SD ± Mean	Max	Min	SD ± Mean	
Height (cm)	186	150	8.31 ± 161.82	188	80	20.22 ± 159.02	0.276
Weight (kg)	100	46	12.59 ± 73.04	100	16	19.37 ± 66.58	0.110
BMI kg/m^2^))	36.80	18.35	4.62 ± 28.10	37.65	12.62	4.80 ± 25.69	0.012
WC* (cm)	122	75	11.92 ± 97.14	117	21	18.88 ± 86.74	0.004
HDL * (mg/dL)	75	29	9.94 ± 49.52	78	34	9.58 ± 50.70	0.746
FBS (mg/dL)*	192	77	22.51 ± 100.16	295	76	39.56 ± 108.62	0.089
TG (mg/dL)*	435	51	84.06 ± 176.22	258	31	62.69 ± 125.32	0.001
SBP (mm Hg)*	170	100	13.85 ± 118.60	190	80	18.67 ± 120.60	0.677
DBP (mm Hg,)*	100	60	7.27 ± 80.40	130	60	11.37 ± 78.20	0.080

*Metabolic syndrome parameters.

BMI: body mass index, WC: waist circumference; TG: triglyceride; HDL: a high-density lipoprotein; FBS: fasting blood sugar; SBP: systolic blood pressure; DBP: diastolic blood pressure.

**Figure 2:**
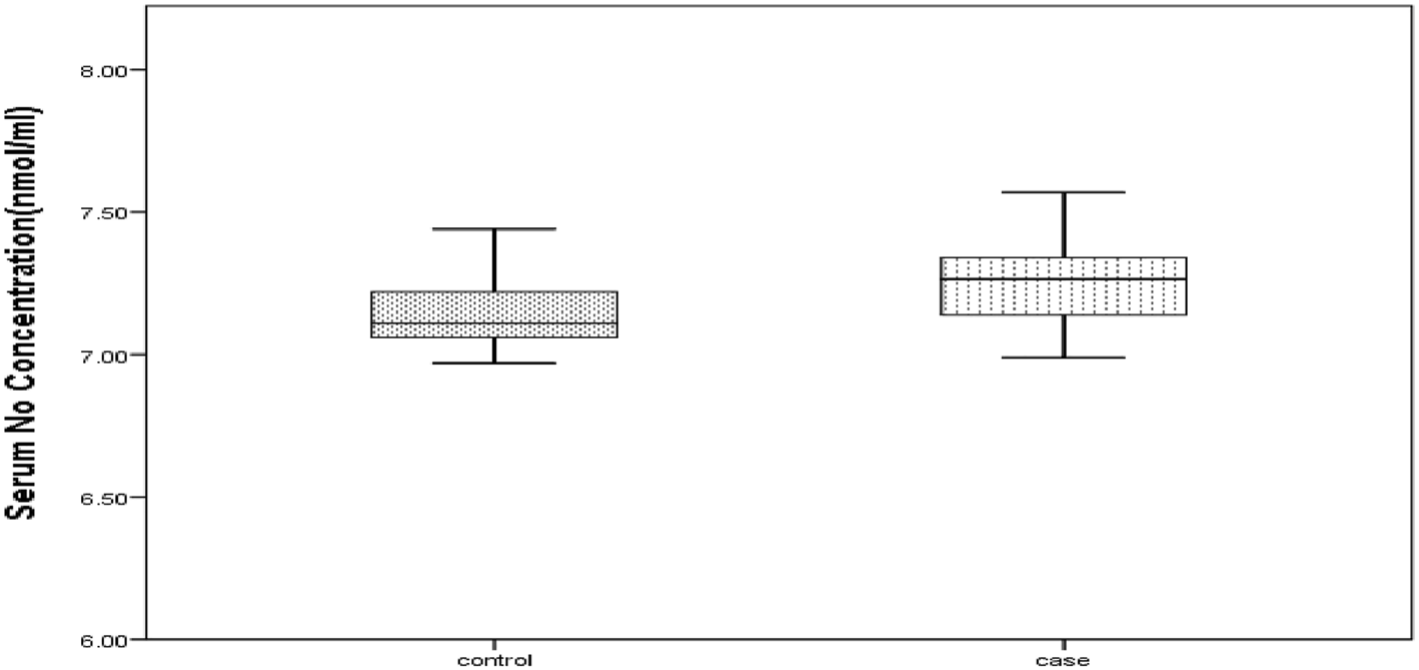
Box plot of serum NO level distribution in the case and control groups with thyroid disorders.

As can be seen, the mean serum NO concentration in the case and control groups was 7.26 ± 0.14 and 7.14 ± 0.12, respectively; there was a statistically significant difference between the case and control groups. Figure 2 shows the distribution of serum NO levels in the case and control groups as a box plot.

Bagheri et al. concluded that serum NO_x_ levels were associated with free T4 in men and anti-TPO in women. In this study, no significant relationship was observed between NO_x_ and TSH^40^. Zoran et al.'s study, which was performed on 88 women (including 44 hypothyroid patients and 38 healthy women), found that serum NO levels in both groups could change and could also be a good predictor of the severity of hypothyroidism^36^.

Verma et al. estimated the nitric oxide of level on the 50 hypothyroid, 50 hyperthyroid, and 50 healthy individuals with a grease reaction. Nitric oxide concentrations were significantly lower in hyperthyroid patients compared with the controls, while this rate was significantly increased in hypothyroid patients. They also suggested that estimating nitric oxide levels in thyroid disorders may help understand the pathogenesis of thyroid disorders^41^. Abhinav Kumar al. revealed that NO levels were significantly reduced in hypothyroidism patients^42^. Mohammed et al. and Kumari et al. also reported a significant association between NO and thyroid disorders^43,44^. On the other hand, Ghazi Saidi et al.'s study,evaluated the correlation between serum nitric oxide concentration and thyroid hormones, and the amount of serum NOx was 30.71 ± 22.17 μmol/L. In this study, like our study, factors affecting serum levels such as BMI, FBS, etc. asked. They concluded that there is no relationship between serum nitric oxide and thyroid hormones^45^.

**Figure 3:**
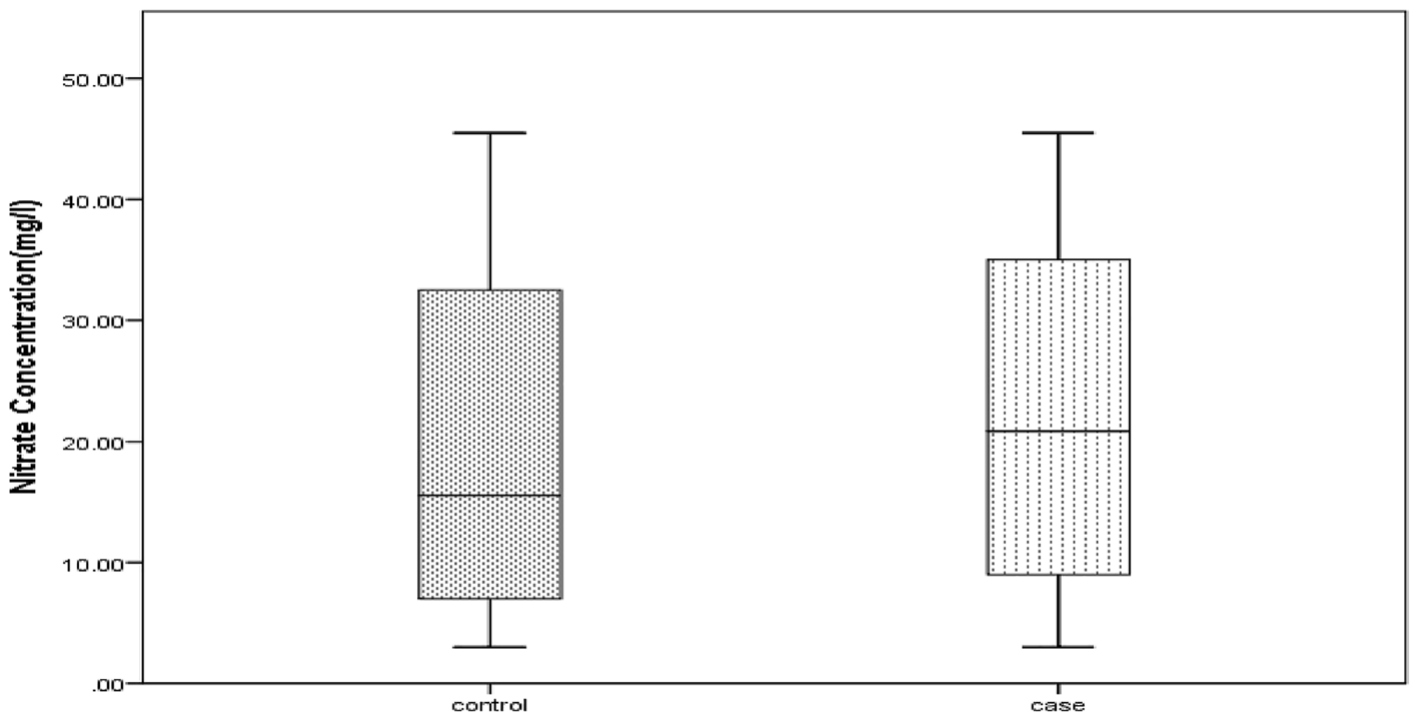
Box plot of serum NO3 level distribution in the case and control groups with thyroid disorders.

As shown in Table 2, the mean NO_3_ concentration of drinking water in the case and control groups was 21.43 ± 13.92 and 20.46 ± 14, respectively. Figure 3 shows a box plot of the distribution of nitrate levels in drinking water in the control group and patients with thyroid disorders.

According to Table 4, the maximum nitrate concentration was 47.5 in the case group and 45.5 mg/L in the control group, which is lower than the nitrate concentration set by the World Health Organization^50^ mg/L^46^. Although the amount of nitrate consumed in all points was less than the standard, there was a big difference between the minimum nitrate (in the patient and control groups equal to 3 mg/L) and the maximum. The supply of distribution systems from different surfaces and groundwater sources can cause differences in nitrate concentration in various places^47^. Examination of water supply showed that areas with higher nitrate concentrations are supplied than groundwater.

**Table 4:** Correlation of the serum NO level with metabolic syndrome components in the case and control groups.

Parameter	People	r	p-value
BMI	Case	−0.207	0.149⁑
	Control	−0.1	0.49⁑
WC (cm)	Case	0.13	0.34*
	Control	0.14	0.32*
HDL (mg/dL)	Case	0.21	0.12*
	Control	−0.12	0.37 *
FBS (mg/dL)	Case	−0.20	0.15*
	Control	−0.13	0.34*
TG (mg/dL)	Case	−0.11	0.41⁑
	Control	−0.03	0.83⁑
SBP (mm Hg)	Case	−0.3	0.31*
	Control	−0.12	0.38*
DBP (mm Hg)	Case	−0.24	0.09*
	Control	−0.01	0.91*
Metabolic syndrome	Case	−0.19	0.16*
	Control	−0.09	0.52*

*Pearson Correlation.

*Spearman Correlation.

Ajdarpour et al. determined the nitrate concentration in 31 sampling points of the distribution system in the Shiraz and observed that the lowest and highest two-year mean concentrations were 2.1 and 58.35; they also stated that nitrate concentration in moment concentration in some places in Shiraz water distribution system was more than the standard level^48^. Another issue with nitrate entering the human body is the nitrate in food, which is shown in Table 1. Typically, about 85% of dietary nitrate (mineral nitrate anion, NO_3_^−^) is obtained from vegetables, and the rest from drinking water^49^. Vegetables can be a significant source of nitrate entering the body of the study participants, and it may be possible that the fertilizer^1^ and water used in vegetables are high in nitrate.

In a study conducted in 2006 by Maria Tajtakova et al., which examined the students in an area with nitrate-contaminated well water sources, thyroid volume and symptoms of thyroid disorders were assessed. Excessive consumption of nitrate in water and their home meals led to an increase in thyroid volume and an increase in the frequency of symptoms of subclinical thyroid disorders^50^.

In the study, Arianna et al. obtained nitrate concentrations in drinking water wells from the California Water Website (GAMA2020). The date of nitrate readings was from 1907 to 2019. They concluded that there was a statistically significant correlation between thyroid cancer registration data and California Water Board data (p < 0.05). They also stated that the rate of thyroid cancer in deprived areas was twice that in non-deprived ones. The number of wells and nitrate-contaminated sites was higher than the state average there^51^.

The relationship between drinking water nitrate concentration and serum nitric oxide in patients with thyroid disorders, (P-value = 0.062) (r = 0.266) and also in the control group, (P-value = 0.756) (r = 0.045) showed a positive correlation although this relationship was not statistically significant.

### Comparison of serum NO correlation and components of metabolic syndrome in the case and control groups

According to Table 3, the results of metabolic syndrome components using clinical examinations for the case and control groups showed a significant difference in BMI, waist size, and triglyceride between the case and control groups (p-value < 0.05).

In a study carried out by Fontenelle et al. on the mechanism of thyroid function in human obesity, it was concluded that the thyroid function of obese individuals could contribute the worsening of metabolic complications and disease progression in the thyroid gland^18^.

Aleksander Kuś et al. in a study on the effect of changes in normal thyroid function on cholesterol levels, blood pressure, and the risk of type 2 diabetes found some changes in normal thyroid function on the serum cholesterol levels, blood pressure, and risk of type 2 diabetes^17^. A study was done by Rizos et al. in 2011 on the effects of thyroid dysfunction on lipid profile; it was concluded that thyroid dysfunction should be considered when evaluating and treating patients with hyperlipidemia^52^. As in our study, the mean triglyceride in patients was higher than that in the case group. According to NCEP-ATP III criteria, TG ˃ 150 can be one of the components of metabolic syndrome.

Table 4 shows the correlation between the serum NO level and metabolic syndrome components in the case and control groups. As shown, there was a correlation between the serum nitric oxide concentration and systolic blood pressure in the case group (P-value = 0.031, r = −0.30) and the control group (P-value = 0.38, r = −0.12). This correlation was statistically significant (p-value < 0.05).

The relationship between serum nitric oxide concentration and waist circumference in the case group (P-value = 0.34, r = 0.13) and control group (P-value = 0.32, r = 0.14) showed a positive correlation, which was not statistically significant. Also, a positive correlation was shown between the serum nitric oxide concentration and HDL level in patients (P-value = 0.12, r = 0.21). This correlation was not statistically significant. There was a negative correlation between serum nitric oxide concentration and HDL level in the control group (r = −0.12, P-value = 0.3) which was not statistically significant.

Correlation between the serum nitric oxide concentration and metabolic syndrome (according to NCEP-ATP III criteria) in patients (P-value = 0.16, r = −0.19) and in the control group (P-value = 0.52, r = −0.09) was negative; this relationship was not statistically significant.

Ashok Kumar et al.'s study included 50 Mets and 50 control. The Mets group was divided into two groups based on the presence or absence of SCH. In this study, a statistically significant correlation was shown in the BMI, systolic and diastolic blood BP, Glucose, HDL, and NO. They also stated that Met S cases should be screened for SCH and treated appropriately^53^.

In our study, the prevalence of Mets was higher in the patient group, but it showed a significant relationship with NO only in patient's diastolic blood pressure.

Hashemi et al. measured the levels of fasting blood glucose (FPG), lipid profile components, and nitric oxide by spectrophotometry on 50 women (25 healthy groups and 25 hypothyroid patients). They found that nitric oxide was significantly different from LDL in the two groups, but it was not significantly different for other components^54^.

In general, the limitation of this study was the small sample size that could not show a cause-and- effect relationship. Therefore, it is recommended that further studies should be done with large sample size. Then, in addition to measuring the water nitrate consumption of the samples, the nitrate of food, especially the vegetables, should be examined.

## Conclusion

The results showed that serum NO was significantly different between the control and case groups (p-value < 0.001). The mean NO_3_ concentration of drinking water in the cases and controls was 21.43 ± 13.92 and 20.46 ± 14, respectively. There was a positive correlation between the concentration of nitrate in drinking water and serum nitric oxide in cases and controls; however, this relationship was not statistically significant. There was a statistically significant relationship between serum nitric oxide and systolic blood pressure in cases (p-value < 0.05). According to NCEP-ATP III criteria, the prevalence of MetS was 34% in the control group and 46% in the case group with a thyroid disorder, which did not show a significant difference between the two groups (p value > 0.05).

## Data Availability

All the data produced and analyzed is available with the Mansooreh Dehghani corresponding author “Email: mandehghani@yahoo.com” and shall be provided on a reasonable request.
